# Challenges on off label medicine use

**DOI:** 10.1016/j.rppede.2015.12.007

**Published:** 2016

**Authors:** Marisa Lima Carvalho

**Affiliations:** Instituto Adolfo Lutz, São Paulo, SP, Brazil

After the Second World War, the so-called pharmacological explosion took place, leading to major advances in the treatment of diseases that were once inevitably fatal or disabling.

This pharmacological expansion contributed to the occurrence of catastrophic incidents, such as the phocomelia epidemic attributed to thalidomide. Since then, concerns about drug safety have contributed to the development and application of clinical and epidemiological methods to assess the benefits and potential risks of any type of therapeutic intervention, whether pharmacological or not.^1^

Indisputably, the administration of a medication aims to obtain a beneficial effect for those who take it. Nonetheless, it is important that the assumptions arising from the analysis of scientific evidence are not forgotten: firstly, some drugs do not have the desired efficacy, and secondly, regardless of their beneficial effects, every medication may produce undesired effects.

When a drug is launched on the market, all the knowledge about it is based on pre-marketing studies: during the development of the molecule, experimental studies on its effects and toxicity are conducted in animals (pre-clinical studies). If no unacceptable toxic effects are observed, the first clinical trials in humans are conducted. These are termed phase I, II, and III studies, which investigate aspects of the pharmacokinetics, toxicity, and efficacy in humans.

In clinical trials, several factors may interfere with the results, such as inclusion and exclusion criteria, sample sizes, and even “apparently ethical” criteria, which, while fully justified in the early stages of the assessment of a new drug, preclude scientific study in certain populations. For a long time, with some exceptions, children have been excluded from clinical trials. Only in phase IV (post-marketing) are the drugs used in children, which may lead them to become the subjects of uncontrolled clinical practice.^1^^,^ 2

This practice of pediatric prescription without clinical evidence, in situations that are different from those studied and advocated (indications, dosages, extemporaneous formulations, age group in which tested), is known as off-label use, which has been demonstrated to be associated with an increase in adverse effects^3^^–^ 5 and should be discouraged.

In this issue of the Revista Paulista de Pediatria, Gonçalves and Heineck conducted a cross-sectional study, with a simple methodology.^6^ In their study, the authors demonstrated that, of the total, 232 (31.7%) prescriptions were off-label, and the following types and frequency were observed: off-label dose – 90 (38.8%); age – 73 (31.5%); and administration frequency – 68 (29.3%). The greatest concern was the finding of overdose of medications whose use in this situation may be fatal, such as salbutamol.

In Brazil, off-label prescription in pediatrics is a frequent practice. Is this practice necessary? What can be done to ensure the safety of children?

In order to protect the health of children and to ensure that medications are used in a more ethical way, in 2007 the European Union issued legislation for the development and authorization of pediatric drugs.^7^ Since then, pharmaceutical companies have been required to develop their medicines both for the adult and pediatric populations, aiming to adapt the drug to the needs, dosage, dosage form, and administration route, among others, in order to ensure effectiveness and that safety is not affected by the risk of overdose. A Pediatric Committee was also created to evaluate the pediatric investigation plans (PIPs) presented by pharmaceutical companies. The committee consists of 12 representatives of the member states; among its functions, the elaboration of an inventory of specific pediatric needs is noteworthy.^7^

Also in 2007, the World Health Organization (WHO) published the first list of Essential Medicines for Children, which is reviewed every 2 years, and launched the “make medicines child size” campaign, in order to raise awareness and promote a global action on the problem of lack of pediatric formulations.^8^

In 2012, under the Investigational New Drug (IND) program, the Food and Drug Administration (FDA), the regulation agency of the United States, created the Safety and Innovation Act (FDASIA-2012), which established the Pediatric Study Plan. This plan is required for new molecules, new indications, new dosage forms, new dosages, and new administration routes.^9^^,^ 10

In Brazil, there are isolated initiatives by healthcare institutions that, by standardizing drugs and creating pharmacology committees, among other measures, are able to evaluate the off-label use of drugs. In the state of São Paulo, the Health Surveillance Center (Centro de Vigilância Sanitária [CVS]) acts in the pharmacovigilance area based on the reports of adverse events, publishing Therapeutic Alerts on Pharmacovigilance in the Official Journal. The CVS has recently published two alerts, “Methylphenidate: indications and adverse reactions” (July 2013) and “Risk of pancreatic cancer associated with incretin-based therapy” (February 2014). Both are focused on alerting, following adverse reactions from the off-label use of drugs. The first is widely used in children.^11^^,^ 12 At the federal level, the Collegiate Board Resolution (Resolução da Diretoria Colegiada [RDC]) No. 9, of 20 February 2015, which aims to establish the procedures and requirements for the conduction of clinical drug trials, indicated that post-marketing clinical trials are subject only to the Notification of Clinical Trial.^13^

Above all, to foster ethical off-label drug use, it is necessary that this exceptional use is clinically justified, even if it is accompanied by clarification and consent of the parents or guardians.^14^ This measure can be taken by healthcare facilities. The Brazilian National Health Surveillance Agency (Agência Nacional de Vigilância Sanitária [Anvisa]), following the example of the regulatory body of the European Union, should establish criteria and standards that stimulate comparative studies and demonstrate the efficacy and safety of medication use in children. When promising, therapies should be tested in controlled clinical trials and their package inserts should be reformulated.
