# Supplementary material for: Challenges on off label medicine use
Source: Rev Paul Pediatr. 2016 Jan-Mar;34(1):1–2. doi: 10.1016/j.rppede.2015.12.007 (PMC4795713; doi:10.1016/j.rppede.2015.12.007)
Supplement: Translation

# Translation: O desafio do uso *off label* de medicamentos

**Authors:** Marisa Lima Carvalho

**Affiliations:** Instituto Adolfo Lutz, São Paulo, SP, Brasil

Após a Segunda Guerra Mundial ocorreu o que se costuma chamar de explosão farmacológica, que deu lugar a grandes avanços no tratamento de enfermidades que antes eram inevitavelmente mortais ou incapacitantes.

Essa expansão farmacológica contribuiu para a ocorrência de acidentes gravíssimos, como a epidemia de focomelia atribuída à talidomida. Desde então, a preocupação com a segurança dos medicamentos contribuiu para o desenvolvimento e a aplicação de métodos clínicos e epidemiológicos para avaliar os benefícios e os riscos potenciais de qualquer tipo de intervenção terapêutica, seja farmacológica ou não.^1^

É claro que com a administração de um medicamento pretende-se obter um efeito benéfico para quem o toma. Não obstante, é importante que as premissas que derivam de análises de evidências científicas não sejam esquecidas: em primeiro lugar, alguns medicamentos não têm a eficácia pretendida e, em segundo, independentemente de seus efeitos benéficos, todos os medicamentos podem produzir efeitos não desejados.

Quando um medicamento é lançado no mercado, todo o conhecimento sobre o fármaco baseia-se nos estudos pré-comercialização: durante o desenvolvimento da molécula, iniciam-se estudos experimentais sobre seus efeitos e sua toxicidade em animais (estudos pré-clínicos) e, caso não se observem efeitos tóxicos inaceitáveis, fazem-se os primeiros ensaios clínicos em humanos. São os denominados estudos de fase I, II e III, para investigar aspectos relacionados com a farmacocinética, toxicidade e eficácia em seres humanos.

Nos ensaios clínicos vários fatores podem interferir nos resultados, como os critérios de inclusão e exclusão, o tamanho das amostras e até mesmo critérios “aparentemente éticos”, que, embora plenamente justificados nas primeiras fases de avaliação de um novo fármaco, impedem o estudo científico em certas populações. Durante muito tempo, salvo exceções, as crianças foram excluídas dos ensaios clínicos. Apenas na fase IV (pós-comercialização) os medicamentos passam a ser usados em crianças, o que pode favorecer que se tornem sujeitos de uma prática clínica não controlada.^1^^,^ 2

Essa prática de indicação pediátrica sem evidências clínicas, em condições diversas das estudadas e preconizadas (indicações, posologias, formulações extemporâneas, idade em que foi testada), são conhecidas como uso *off label*, que está comprovadamente associado a um aumento dos efeitos adversos^3^^-^ 5 e deve ser desencorajado.

Neste fascículo da Revista Paulista de Pediatria, Gonçalves e Heineck fizeram um estudo descritivo transversal, com metodologia simples.^6^ Neste estudo os autores mostraram que, do total, 232 (31,7%) prescrições eram *off label* e observaram-se os seguintes tipos e frequências: *off label* de dose - 90 (38,8%); de idade -73 (31,5%); e de frequência de administração - 68 (29,3%). O mais preocupante foi o achado de sobredose para medicamentos cujo uso nessa situação pode ser fatal, como é o caso do salbutamol.

A indicação de uso *off label* de medicamentos em pediatria no Brasil é uma prática recorrente. Será que essa prática é necessária? O que se pode fazer para garantir a segurança das crianças?

Com o objetivo de proteger a saúde das crianças e garantir que esses medicamentos sejam usados de forma mais ética, desde 2007 a União Europeia editou uma legislação para o desenvolvimento e a autorização de medicamentos pediátricos.^7^ Desde então as indústrias farmacêuticas estão obrigadas a desenvolver seus medicamentos tanto na população adulta como na população pediátrica, com o objetivo de adaptar os medicamentos às necessidades, à posologia, à forma farmacêutica, à via de administração, entre outros, a fim de que sejam eficazes e sua segurança não seja alterada pelo risco de superdosagem. Foi criado ainda um Comitê Pediátrico, encarregado de assegurar a avaliação dos Planos de Investigação em Pediatria, os PIPs, apresentados pelas indústrias farmacêuticas. O comitê é composto por 12 representantes dos países membros e entre suas funções se destaca a elaboração de uma lista que contemple as necessidades específicas em Pediatria.^7^

No mesmo ano de 2007, a Organização Mundial de Saúde (OMS) publicou a primeira Lista de Medicamentos Essenciais para Crianças, que é revisada a cada dois anos, e lançou ainda a campanha *Make medicines child size*, com o intuito de sensibilizar e promover uma ação global sobre o problema da falta de formulações pediátricas.^8^

Nos Estados Unidos da América o órgão regulador, Food and Drug Administration (FDA), criou em 2012, subordinado ao IND (Investigação de Novas Drogas), o Safety and Innovation Act (FDASIA-2012), que implantou o Plano de Estudos Pediátricos. Esse plano é exigido para novas moléculas, nova indicação, nova forma farmacêutica, nova posologia e nova via de administração.^9^^,^ 10

No Brasil existem iniciativas isoladas de estabelecimentos de saúde que, ao padronizar medicamentos, criar comissões de farmacologia e outras medidas, conseguem avaliar o uso de medicamentos *off label*. No Estado de São Paulo, O Centro de Vigilância Sanitária (CVS), na área de farmacovigilância, tem por base as notificações de eventos adversos e faz publicar em Diário Oficial Alertas Terapêuticos em Farmacovigilância. Recentemente o CVS publicou dois Alertas, “Metilfenidato: indicações terapêuticas e reações adversas” (julho de 2013) e “Risco de neoplasia pancreática associado à terapia baseada nas incretinas” (fevereiro de 2014). Ambos têm como foco o alerta após reações adversas decorrentes do uso *off label* dos medicamentos. O primeiro é amplamente usado para crianças.^11^^,^ 12 No âmbito federal, a Resolução da Diretoria Colegiada (RDC) n° 9, de 20 de fevereiro de 2015, que tem por objetivo definir procedimentos e requisitos para a feitura de ensaios clínicos com medicamentos, ressalta que os ensaios clínicos pós-comercialização estão sujeitos apenas a Notificação de Ensaio Clínico.^13^

De imediato, para induzir o emprego ético de medicamentos *off label*, é necessário que seu uso excepcional seja justificado clinicamente, ainda que seja acompanhado de esclarecimento e consentimento dos responsáveis.^14^ Essa medida pode ser tomada pelas unidades de saúde. A Agência Nacional de Vigilância Sanitária (Anvisa), a exemplo do órgão regulador da União Europeia, deveria estabelecer critérios e normas que induzam estudos comparativos e mostrem a eficácia e segurança do uso de medicamentos em crianças. Quando promissoras, as terapias devem ser testadas em ensaios clínicos controlados e as bulas reformuladas.
